# The mTST – An mHealth approach for training and quality assurance of tuberculin skin test administration and reading

**DOI:** 10.1371/journal.pone.0215240

**Published:** 2019-04-17

**Authors:** Saeedeh Moayedi-Nia, Leila Barss, Olivia Oxlade, Chantal Valiquette, Mei-Xin Ly, Jonathon R. Campbell, Zhiyi Lan, Placide Nsengiyumva, Federica Fregonese, Mayara Lisboa Bastos, Danielle Sampath, Nicholas Winters, Dick Menzies

**Affiliations:** 1 McGill International TB Centre, McGill University, Montreal, QC, Canada; 2 Department of Epidemiology Social Medicine Institute, State University of Rio de Janeiro, Rio de Janeiro, Brazil; University of New Mexico Health Sciences Center, Division of Infectious Diseases, UNITED STATES

## Abstract

**Background:**

The Tuberculin Skin Test (TST) is a relatively simple test for detecting latent tuberculosis infection (LTBI) but requires regular quality assurance to ensure proper technique for administration and reading. The objective of this study was to estimate the accuracy and reproducibility of an mhealth approach (**the mTST**) to measure the size of swelling immediately following TST administration (TST injection bleb) and after 48–72 hours (TST induration).

**Methods:**

Five non-clinical and one clinical reviewer measured the size of TST injection blebs, and TST indurations using smartphone acquired photos of sites of TST administration and readings in patients, or saline injections in volunteers. The reference standard was the onsite measurement (measured by an experienced TB nurse) of the actual TST injection bleb, or induration. Agreement of reviewers’ measurements with the reference standard, as well as agreement within and between reviewers, was estimated using Cohen's kappa coefficient.

**Results:**

Using the mTST method to assess bleb size in 64 photos of different TST injections, agreement between reviewers, and the reference standard was very good to excellent (κ ranged from 0.75 to 0.87), and within-reviewer reproducibility of readings was excellent (κ ranged from 0.86 to 0.96). Using the mTST method to assess TST induration in 72 photos, reviewers were able to detect no induration (<5mm) and induration of 15mm or greater with accuracy of 95% and 92% respectively, but accuracy was only 20% and 77% for reactions of 5-9mm and 10-14mm respectively.

**Conclusion:**

The mTST approach appears to be a reliable tool to assess TST administration. The mTST approach was accurate to read indurations of 0-4mm or 15+mm, but less accurate for reactions of 5-14mm. We believe the mTST approach could be useful for training and quality assurance in locations where on-site supervision is not possible.

## Introduction

Based on recent estimates, approximately 1.7 billion people worldwide have latent tuberculosis infection (LTBI)[1]. Among this vast population, an estimated 10% (170 million people) will develop active tuberculosis (TB) over their lifetime. Testing and treatment of people with LTBI has been shown to significantly reduce the risk of progression to active TB[2]. Testing for LTBI is an important element of LTBI management. Either a tuberculin skin test (TST) or interferon gamma release assay (IGRA) may be used for diagnosis[3]. The TST is often the only method available, particularly in low and middle-income countries (LMIC) where the incidence of active and latent TB is highest[4,5]. However, incorrect administration technique has been cited as a potential cause for false negative TST results[6], and poor reading technique may also result in incorrect results. While the techniques for TST administration and reading are relatively simple, training and supervision are needed to establish and maintain proficiency, as for any diagnostic test.

The World Health Organization (WHO) recommended the development and testing of digital tools to support LTBI programmatic management [7]. Smartphones are one such potential tool, as they are increasing in accessibility, user-friendly, and can provide high-definition images, which can easily be transmitted, allowing for evaluation and feedback from experts located at a distance [8]. Smartphone ownership has increased substantially in LMIC over the last several years [9]. It is estimated that by 2025, 71% of the world population will own a smart phone, with the largest increases in low- and middle-income regions[10]. Digital images shown on smartphones have been used to support health care worker training for cervical cancer screening, post-operative wound monitoring, and detecting melanoma [11–13]. To date, two studies have evaluated smartphone images of *simulated* TST indurations and found that these images could be used to accurately measure induration size [14,15]. No study has evaluated this technology when used to improve actual tuberculin test administration or reading in humans using the mobile TST (mTST) defined as use of photos of a TST injection site taken by health care worker immediately after the tuberculin injection and evaluated remotely by an experienced reviewer.

The aim of this study was to estimate the accuracy and reproducibility of the mTST using independent measurements by off-site reviewers of the swelling (‘bleb’) produced immediately after a real TST injection, and of TST indurations in patients, using photos taken with a smartphone and transmitted to the reviewers.

## Methods

### Establishing the best technical method for taking photos using a smartphone

In preliminary work, we assessed the technical specifications required to take a high-quality photo using a smartphone of the injection bleb immediately after TST administration and of the induration after 48–72 hours. Multiple variations of smartphone brands (ranging in size from 4.8 inches to 6.5 inches in length), positioning, angles, distance from injection site or induration, lighting, measurement scale, and transmission of photos were tested to determine the optimal conditions and specifications for taking a photo of the injection bleb and the induration. Photos acquired using various techniques were reviewed in order to select the optimal specifications needed to produce a high-quality image. We found that the type or size of cellphone used did not affect the quality of photos or the methods used. We also found that the quality of images taken using a smartphone was sufficient when low definition images were used. Other technical specifications are summarized in an instruction manual and accompanying video developed to describe the mTST method. (See S1 Appendix: “mTST Instructions—Health Care workers”, and the video at the following link: https://youtu.be/S8gLaIPqvho).

**Fig 1:**
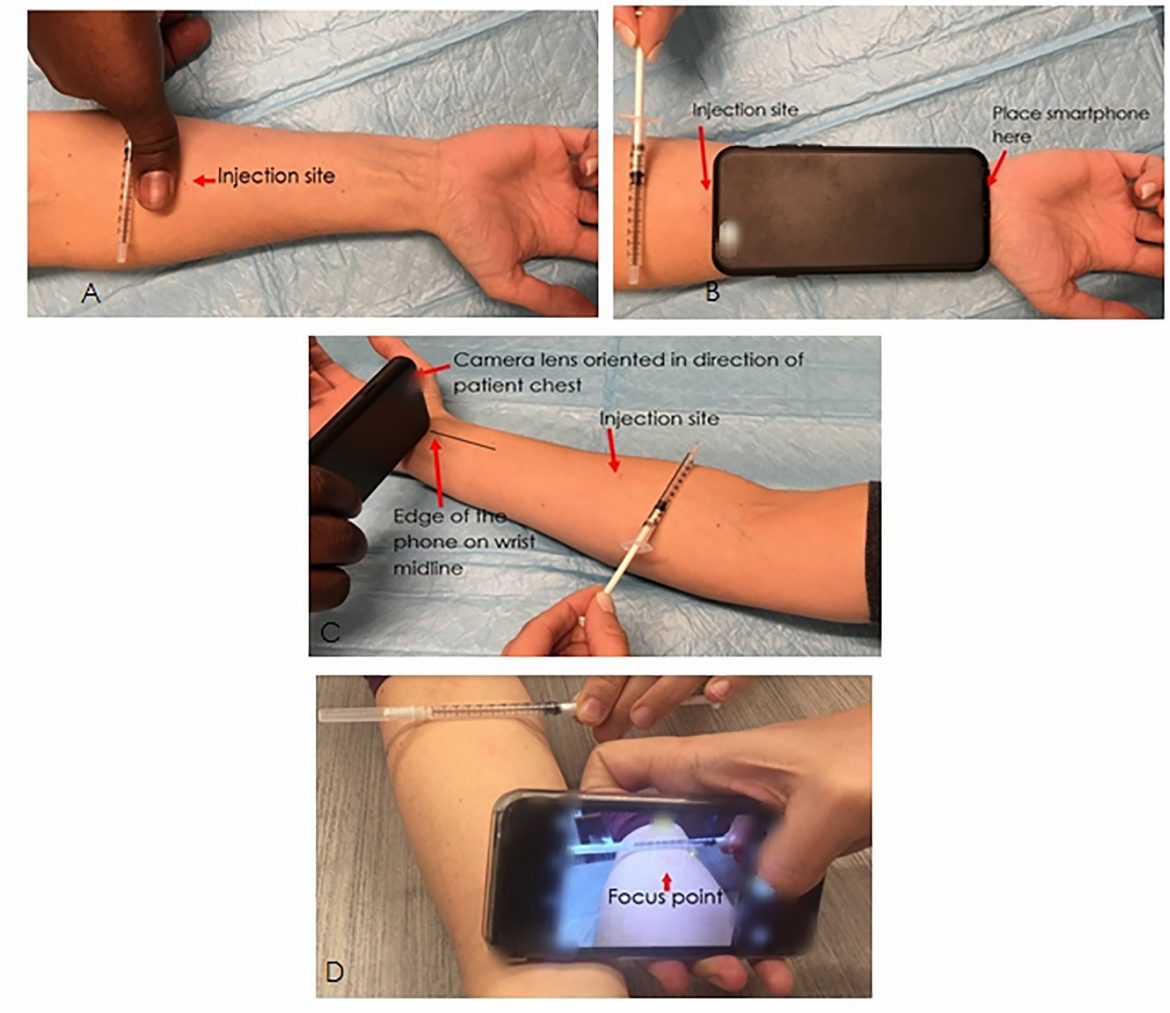
Method to photograph the tuberculin injection site. A: Position the syringe proximal to TST injection site on forearm, at a distance equivalent to the width of a thumb. B: Position the smartphone distal to the injection site, at a distance equivalent to the length of the smartphone. C: Turn flash ON and hold the smartphone positioned so that the camera lens is at the top edge of the phone (and not against the arm), and pointed towards the tuberculin injection site. D: Tap the screen to focus on the TST injection site. Take at least 3 photos.

Fig 1 illustrates the four main steps to take a high-quality photo of the tuberculin injection site. Examples of photos of TST injection which are adequate (good) quality and inadequate (poor) quality are shown in Fig A of S2 Appendix.

**Fig 2:**
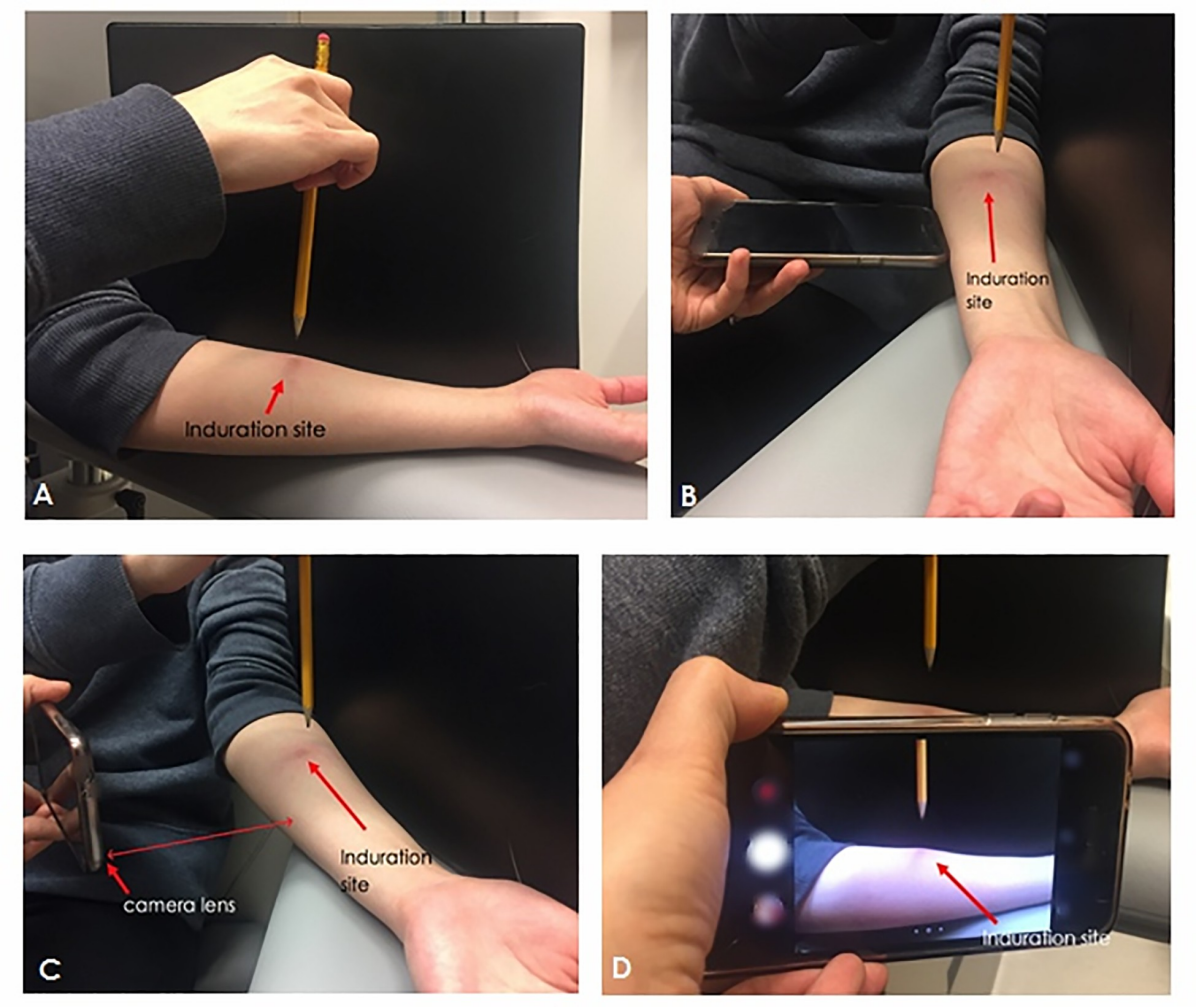
Method to photograph TST induration. A: Position the patient’s arm in-front of a black background. A pen or pencil should be held 2 cm above the site where the TST injection was given. B: Position the smartphone approximately one smartphone distance (lengthwise) from the patient’s forearm. C: Turn flash OFF and hold the smartphone positioned so that the camera lens is at the bottom edge of the phone (closer to arm) and pointed towards the induration. D: Tap the screen to focus on the induration site. Take at least 3 photos.

The four main steps describing how to take a photo of the TST induration 48–72 hours after tuberculin injection are shown in Fig 2, and example photos of four different induration sizes in Fig B in S2 Appendix.

### Obtaining reference standard measurements and taking the on-site mTST photos

Consecutive patients over the age of 18 years who underwent a TST for clinical indications at the Montreal Chest Institute outpatient respiratory clinic in Montreal, Quebec, Canada, between August 1, 2017 and July 31, 2018 were potentially eligible to participate in this study. mTST photos of TST administration and/or induration were taken if the patients provided verbal agreement. A sample size was calculated to determine the total number of participants needed in the study, however this was not done for specific subgroups (ie. by reaction size for induration readings). In order to obtain a sufficient number of participants within different groups we estimated the level of precision that was required around estimates and continued to add participants until we reached that level of precision.

One of two TB nurses, each with over ten years experience in TST administration and reading, performed TST administration using the Mantoux technique. This consisted of an intradermal injection of 0.1 ml (5 tuberculin units) of purified protein derivative (PPD) on the inner aspect of the forearm, approximately 10 cm distal to the elbow[6]. To simulate incorrect TST administration, we used the standard TST administration technique to administer normal saline with volumes of 0.05 ml to healthy volunteers. Measurement of the size of the ‘bleb’ resulting immediately after injection of this inadequate amount of test material was used to decide on a cut-point to determine if the injection was performed with ‘correct’ technique.

The reference standard for TST injection site ‘bleb’ size was the measurement by the same TB nurse of the bleb immediately following TST administration. The diameter of the injection bleb was measured at its widest transverse diameter (at a right angle to the long axis of the forearm) using a caliper-type ruler and recorded (Fig C in S2 Appendix). mTST photos of the TST injection bleb were taken immediately after the measurement of the injection site bleb.

The reference standard for TST induration was the reading 48–72 hours after TST administration by the same TB nurses. The transverse diameter of the induration was demarcated using the Ballpoint Pen Method [6], measured with a caliper-type ruler, and recorded in mm. Photos of the induration were taken immediately before the onsite measurement of the induration.

### Measurement of mTST photos by reviewers

Five reviewers with no experience in TST administration or reading (*non-clinical reviewers*) were selected. To determine if experience affected accuracy of induration assessment, one experienced nurse (*clinical reviewer*) also reviewed all images of indurations. All reviewers were provided with written instructions that described how to measure the bleb on a digital photo and watched a training video (see S3 Appendix for: “mTST Instructions for Quality Assurance of TST administration”, and video available at: https://youtu.be/DZbuygias7w). Reviewers were provided with a series of smartphone photos in random sequence and were blinded to the on-site measurement of the injection blebs and indurations.

#### Bleb measurement

**Fig 3:**
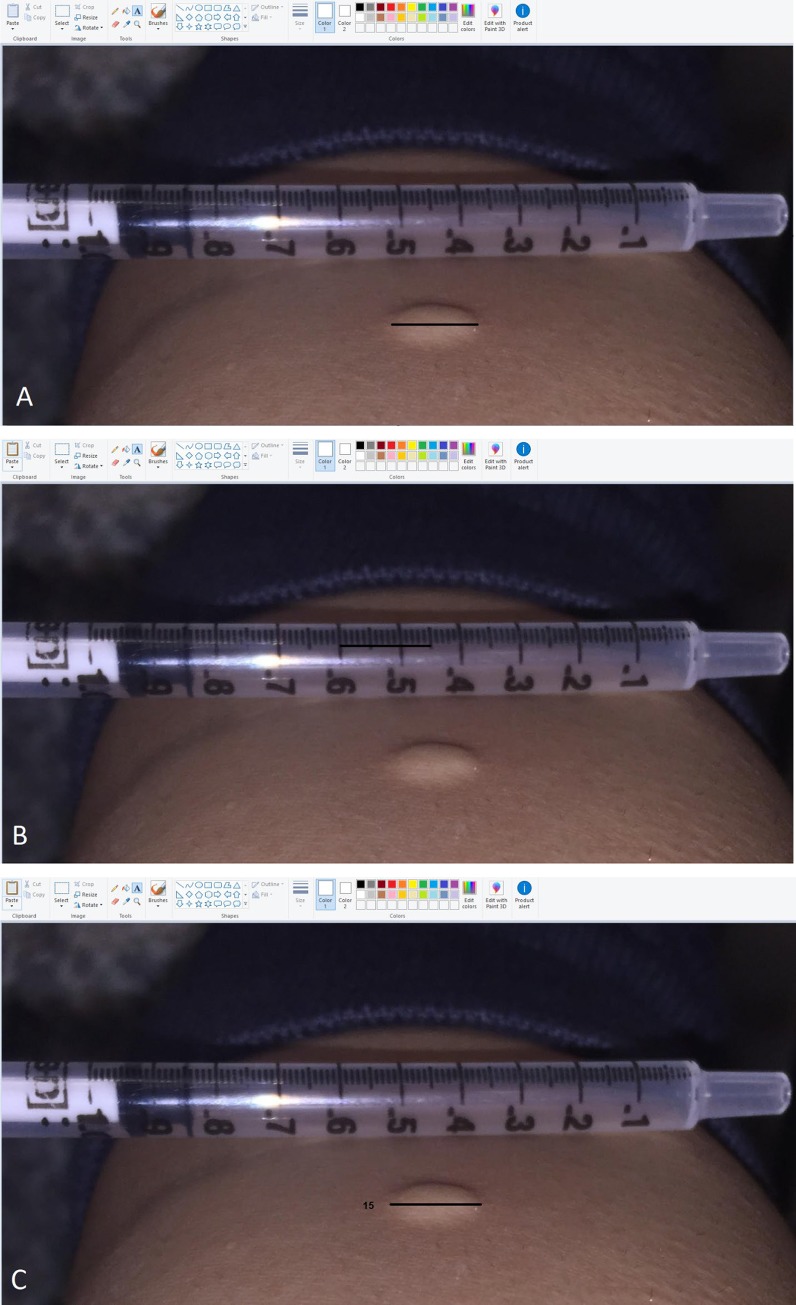
Reading photos of TST injection site. A: Using standard photo editing software (e.g. Microsoft Paint, or Preview), draw a digital line over the bleb at its widest point. B: Move the line to align with tick marks on syringe. C: Record the number of tick marks, and convert the measurement into millimeters.

For the injection bleb measurement, the diameter of the injection bleb at its widest transverse diameter was measured by first drawing a digital line across the bleb using standard photo editing software (e.g. Microsoft Paint or Preview (Mac)) on a computer. The line was then placed next to the tuberculin syringe that appeared within the image, in order to determine the number of tick marks on the tuberculin syringe (Fig 3). The measurement (in number of tick marks) was then converted into millimeters and recorded (see Table E of S3 Appendix). We dichotomized the size of blebs into ‘correct’ and ‘incorrect’, based on the actual amount of test material injected.

#### Induration measurement

The mTST images showing induration were categorized as “induration absent” if no induration could be seen, or “induration present” if any induration could be seen.

#### Intra reader reliability

Reviewers measured each image on two separate occasions, at least two weeks apart, in order to assess intra-reviewer reliability. For the second reading the photos were provided in a different order; reviewers remained blinded to the on-site measurements and were blinded to their original reading.

### Data analysis—Assessment of agreement with reference standard

#### Bleb measurement

**Fig 4:**
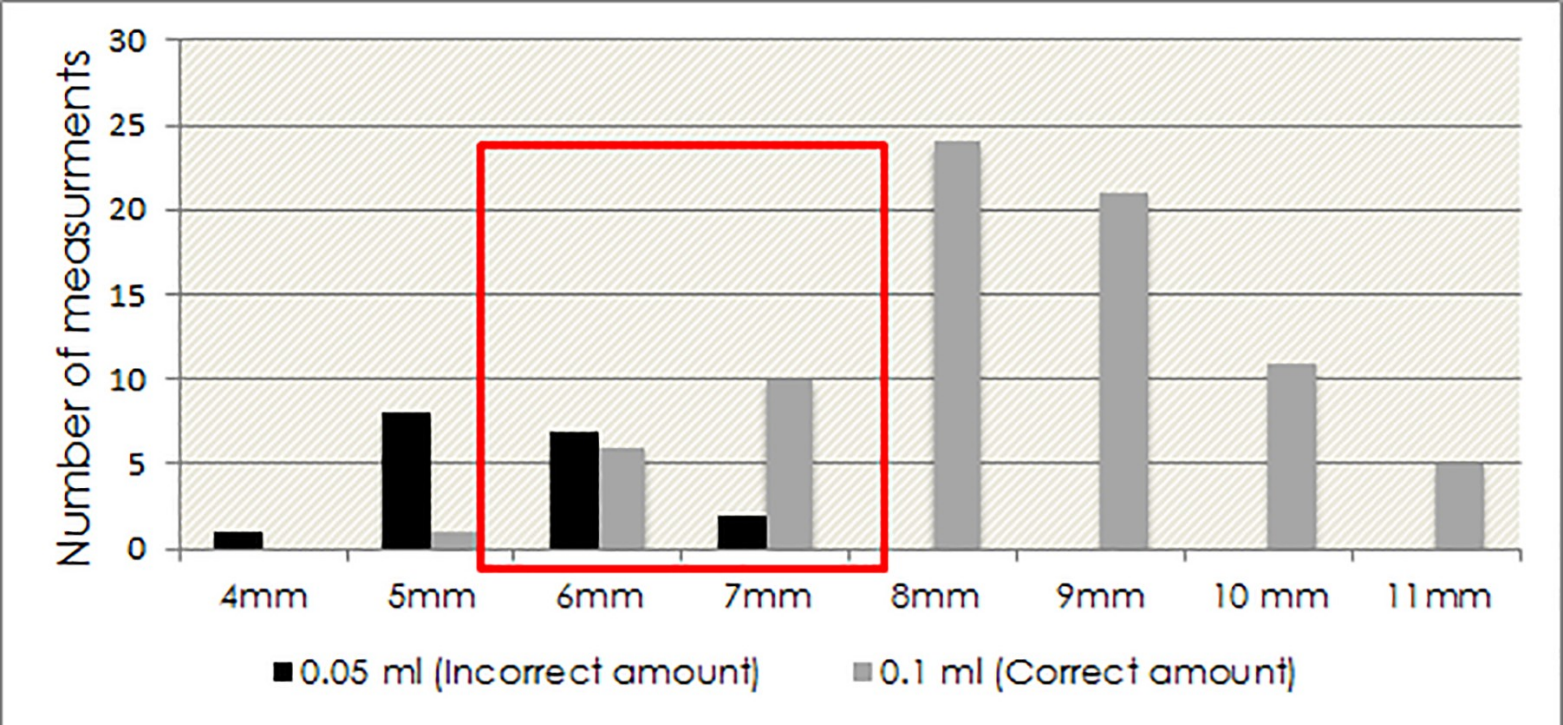
Number of measurements. By bleb size (mm) reported by reviewers, for 78 measurements of blebs generated using the correct volume (0.1ml) of injected material and 18 which used the incorrect volume (0.05ml).

For TST injections, we first had to determine the size of bleb to serve as the optimal cut-point to discriminate correct versus incorrect TST administration based on quantity of material injected. To do this, we examined the frequency distribution of ‘bleb’ sizes following injection of correct amounts of tuberculin or normal saline (0.1ml), and incorrect volume (0.05ml) of saline. A total of 96 bleb measurements were made from 16 TST injections. 78 bleb measurements were on those generated using the correct volume (0.1ml) of injected material and 18 which used the incorrect volume (0.05ml). As shown in Fig 4, 71/78 (91%) of mTST measurements of injections using the correct volume (0.1ml) of test material and only 2/18 (11%) of mTST measurements of injections made using the incorrect volume (0.05ml) of test material were ≥7mm.

Based on this finding, an injection site bleb size of 7 mm was chosen as the optimal cut-point to determine if the correct volume of tuberculin was administered.

This cut point was then used to dichotomize all readings (reference and reviewers) into ‘correct’ or ‘incorrect’ TST administration, allowing estimation of Cohen's kappa coefficient(κ) between reference standard and each reviewer measurement, as well as between and within reviewers.

#### Induration measurement

Reference induration measurements were categorized as follows: 0-4mm, 5-9mm, 10-14mm, and 15+mm. Readings in the ≥5mm categories were considered to have “induration present,”. Within each of the categories the percent of readings in which reviewers correctly reported if an induration was absent or present was calculated. We repeated these calculations after dichotomizing all readings into 0-9mm and 10+mm categories.

## Ethics

Ethical approval for this study was obtained from the research ethics board of the McGill University Health Centre Research Institute (MUHC-REB# 2018–3787). Patients provided verbal agreement to allow the nurse to take a photo of the TST site (administration or reading). The verbal consent procedure used English or French verbal scripts, which were approved by the MUHC-REB. As no patient identifying information was collected, written consent was not deemed to be necessary. A note was placed in the patient’s chart if verbal consent was given to participate in the research study. To maintain confidentiality, no personal or identifying information was collected, and photos were not taken if there were any potentially identifiable information or marks (e.g. tattoos, birthmark) in the area of TST injection. When photos were stored electronically they were labelled only with the date of the photo, and the nurse’s measurements.

## Results

### TST administration bleb

64 photos of TST administration were assessed. Using the 7mm cut-point we determined to best distinguish injection of correct versus incorrect amounts of test material, agreement of measurement from these photos with the reference (on-site) measurement was excellent for three reviewers and substantial for the other two. For all reviewers, near perfect within-subject agreement was observed for duplicate readings (Table 1).

**Table 1:** Agreement within and between each non-clinical reviewer and the reference standard for measurement and classification of TST blebs–judged as correct (7mm or more) or incorrect (less than 6mm).

Reliability	Cohen’s kappa coefficient between reference standard and each reviewer	Cohen’s kappa coefficient within reviewer
Non-clinical Reviewer 1	0.87	0.93
Non-clinical Reviewer 2	0.75	0.96
Non-clinical Reviewer 3	0.83	0.89
Non-clinical Reviewer 4	0.83	0.89
Non-clinical Reviewer 5	0.79	0.86

### TST induration

For the 72 photos of TST indurations, the agreement between the five non-clinical reviewers’ readings and those of the reference standard are shown in Table 2 and Table 3.

**Table 2:** Accuracy of mTST readings by five non-clinical (NC) reviewers, and one clinical-reviewer of 72 different photos of induration, compared to on-site measurement by an experienced TB nurse. Readings categorized into 4 groups based on size of TST induration measured (using reference standard).

	Reference 0–4 mm (N = 12)		Reference 5–9 mm (N = 9)		Reference 10-14mm (N = 6)		Reference 15 or more mm (N = 45)	
	Induration seen		Induration seen		Induration seen		Induration seen	
	No	Yes	No	Yes	No	Yes	No	Yes
**Results of first reading—detailed**								
NC Reviewer 1	12	0	9	0	2	4	9	36
NC Reviewer 2	12	0	5	4	0	6	0	45
NC Reviewer 3	11	1	8	1	2	4	2	43
NC Reviewer 4	10	2	6	3	1	5	2	43
NC Reviewer 5	12	0	8	1	2	4	4	41
Clinical reviewer	10	2	6	3	2	4	4	41
% Correct on 1^st^ reading (NC reviewers)*	95%		20%		77%		92%	
% Correct on 1st reading * (clinical-reviewer)	83%		33%		67%		91%	
**Results of second reading—summary**								
% Correct on 2^nd^ reading (NC reviewers)*	95%		29%		77%		92%	
% Correct on 2^nd^ reading * (clinical-reviewer)	83%		33%		83%		93%	

* Correct means the reviewer reported no induration seen, if the reference standard measurement was 0-4mm. If the reference standard was 5mm or larger than correct means the reviewer reported induration present. Percent correct based on all readings of all reviewers–simple pooling. NC = Non-clinical reviewer

**Table 3:** Accuracy of mTST readings by five non-clinical (NC) reviewers, and one clinical-reviewer of 72 different photos of induration, compared to on-site measurement by an experienced TB nurse. Readings categorized into two size groups based on size of TST induration measured (using reference standard).

	Reference <10 mm (N = 21)		Reference ≥10mm (N = 51)	
	Induration seen		Induration seen	
	No	Yes	No	Yes
**Results of first reading—detailed**				
NC Reviewer 1	21	0	11	40
NC Reviewer 2	17	4	0	51
NC Reviewer 3	19	2	4	47
NC Reviewer 4	16	5	3	48
NC Reviewer 5	20	1	6	45
Clinical-reviewer 1^st^	16	5	6	45
Clinical-reviewer 2^nd^	16	5	4	47
% Correct on 1^st^ reading (NC reviewers)*	89%		91%	
% Correct on 1st reading * (clinical-reviewer)	76%		88%	
**Results of second reading—summary**				
% Correct on 2^nd^ reading (NC reviewers)*	85%		90%	
% Correct on 2^nd^ reading * (clinical-reviewer)	76%		92%	

* Correct means the reviewer reported no induration seen, if the reference standard measurement was <10 mm. If the reference standard was 10mm or larger than correct means the reviewer reported induration present. NC = Non-clinical reviewer. Agreement based on all readings estimated from simple pooling.

When the TST induration measured 0-4mm (“induration absent”), agreement was excellent, averaging 95%. Agreement between reviewers and the reference standard was much worse (20%) for TST reactions in the 5-9mm range but improved to 77% for reactions in the 10-14mm range and to 92% for reactions of 15mm and larger. Interestingly, agreement between the clinical reviewer and the reference standard was similar to the five non-clinical reviewers for all induration sizes.

Intra-observer agreement was also high for the negative TST reactions, and the large reactions, but lower for TST indurations of 5-9mm and 10-14mm (Table 4), for all reviewers (non-clinical and clinical).

**Table 4:** Within-reviewer agreement if induration present or absent on repeated evaluation of mTST photos of TST (performed 1 month apart) by five non-clinical (NC) reviewers and one clinical-reviewer.

**Agreement witdin four categories of TST induration size**					
Reviewer	agreement–witdin categories				Cohen’s kappa coefficient
	0–4 mm	5–9 mm	10-14mm	≥15 mm	
NC-1	100%	100%	83%	93%	0.89
NC-2	83%	78%	100%	100%	0.83
NC-3	100%	67%	83%	96%	0.79
NC-4	83%	89%	83%	93%	0.78
NC-5	100%	100%	83%	93%	0.88
Clinical-reviewer	83%	100%	83%	98%	0.87
**Agreement within two categories of TST induration size**					
Reviewer	agreement–within categories				
	0–9 mm		≥10 mm		
NC-1	100%		92%		
NC-2	81%		100%		
NC-3	86%		94%		
NC-4	86%		92%		
NC-5	100%		92%		
Clinical-reviewer	91%		96%		

Simple agreement within categories of size of TST based on reference (i.e. on-site reading made with Ballpoint pen method by experienced TB nurses), and overall agreement estimated by Cohen's kappa coefficient

**Fig 5:**
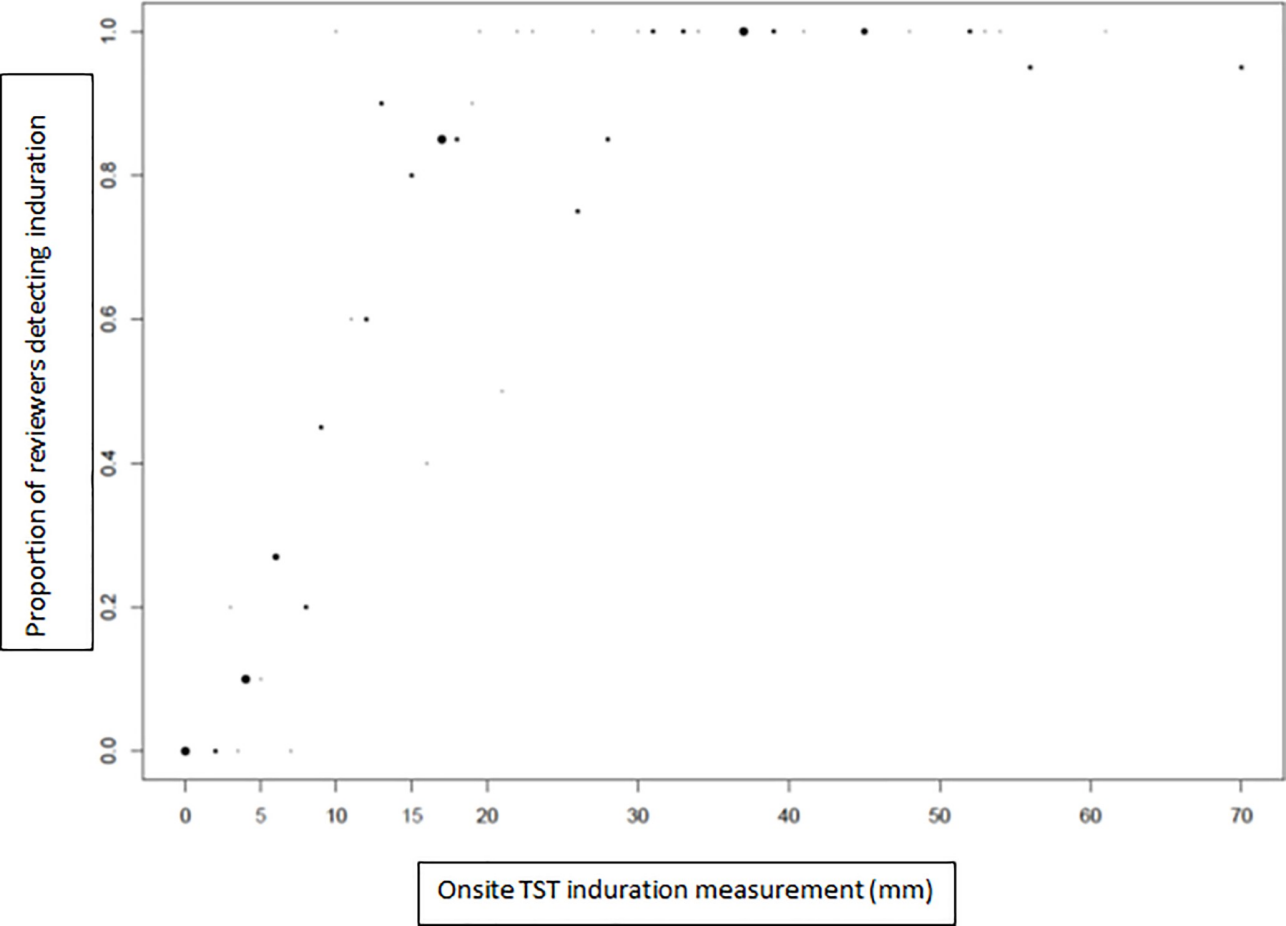
Proportion of reviewers who said that induration was present, by size of TST (mm) measured onsite by TB nurse. Note: The size of the dots is proportionate to the number of readings.

As seen in Fig 5, the likelihood that induration was detected by the reviewers increased in an almost linear fashion, with increasing on-site measured induration size.

## Discussion

In this study we have shown that reviewers using the mTST approach had good accuracy in identifying TST injections of correct and incorrect amounts of test material, and that the interpretation of these photos is highly reproducible, based on between and within-reviewer agreement. Using the mTST approach, reviewers could identify TST indurations less than 5mm and larger than 14mm accurately, compared to the reference standard of on-site measurements by an experienced TB nurse. However, this approach was less accurate for indurations of 5–14 mm. Agreement within and between-reviewers was very good–indicating that reading indurations with the mTST method provided reproducible results.

This study had several strengths. The study was conducted entirely with smartphones that are widely available, increasing the potential applicability of results. The non-clinical reviewers had no prior experience with TST administration or reading, yet after brief training they could achieve reproducibly accurate assessments of TST administration when compared to the reference standard. For assessment of TST induration, results were less accurate, but still very reproducible and not worse than assessments by an experienced clinical reviewer. This suggests our results should be easily reproduced in other settings, even if reviewers have not had extensive prior experience with TST techniques. In contrast to two recent studies [13,14], our study evaluated real TST injections and indurations in patients, making the results more relevant to clinical practice.

Nevertheless our study had important limitations. The number of readings of smaller TST indurations (in the 5-9mm and 10-14mm ranges) were limited–and this proved to be the size of indurations which were the most difficult to read. Future studies with more participants would be helpful to provide more information, especially for the group who had small (5-9mm) and intermediate (10-14mm) reactions. The development of additional technology could also help with readings in the groups that were difficult to read using 2D images alone. Given the inherent variability of human reading skin reactions from digital images, the development of an app that could produce an automated reading with a score could be a helpful next step.

The WHO has called for increased treatment of latent TB in low and middle-income settings(4), and recommended LTBI testing to identify candidates for LTBI therapy, although this is not required for children under 5, and those who are HIV infected [4]. These recommendations also noted that “TST may require significantly fewer resources than IGRA and may be more familiar to practitioners in resource-constrained settings”[4]. The TST has many advantages for large scale implementation in these settings–notably the simplicity of the test as it can be performed almost anywhere without need for lab facilities, and at lower cost than commercial IGRA’s. However, the challenge facing TB programs in resource limited settings is how to provide adequate training and quality assurance for large scale expansion of TST.

As with any diagnostic test, the TST requires proper methods of administration and reading to provide accurate results. Direct observation for ongoing monitoring and quality assurance can be difficult in settings where TST is performed only occasionally, or in remote communities. Taking the photos for mTST is very simple–requiring only a smart phone and the ability to transmit the images. We have shown that reviewing the mTST photos is also simple, easily learned and highly reproducible. In addition to bleb size, the mTST can be used to identify poor injection positioning, excessive bleeding or leakage of tuberculin material. This was not assessed in our study, as this seemed self-evident, and also these errors were not made by the experienced TB nurses in our setting. However, using the mTST to identify these errors could easily be incorporated into training and practise. The mTST could also be helpful to offer recommendations for care of severe reactions such as local blistering. Hence, we believe this method could be useful in low and high incidence settings, for follow-up monitoring and quality control of both TST administration and reading. We have developed several tools for adoption of this method, including training manuals, on-line videos, and training sets of photos of correct and correct TST injections, as well as indurations of varying sizes. These are available on-line, or from authors on request.

## Conclusion

In conclusion, the mTST approach appears to be reliable for assessment of technique of TST injection. Although this method is less accurate to detect small TST indurations in the 5-9mm range, it is much more reliable for larger indurations, particularly indurations of 15mm or larger. We believe the mTST is a simple and easily implemented method that could serve as a useful training and quality control tool in many settings.

## Supporting information

S1 Appendix mTST: For quality assurance of tuberculin skin testing (TST) administration—instructions for healthcare workers: How to take mTST photos. (DOCX)

S2 Appendix Supporting information. (DOCX)

S3 Appendix Guide for reviewers performing the TST quality assurance (i.e. those reviewing the mTST photos taken by health care workers). (DOCX)
