# Supplementary material for: The mTST – An mHealth approach for training and quality assurance of tuberculin skin test administration and reading
Source: PLoS One. 2019 Apr 17;14(4):e0215240. doi: 10.1371/journal.pone.0215240 (PMC6469794; doi:10.1371/journal.pone.0215240)
Supplement: S1 Appendix — (DOCX) [file pone.0215240.s001.docx]

These instructions describe how to take a mobile health photo of a tuberculin skin test or TST (mTST) injection site immediately after injection, and how to email the photo for review. *(Instructions prepared by: Saeedeh Moayedi-Nia, Leila Barss, Olivia Oxlade & Dick Menzies at the McGill International TB Centre. If any questions please contact us by email:* [*Olivia.Oxlade@McGill.ca*](mailto:Olivia.Oxlade@McGill.ca)*, or Dick.Menzies@McGill.ca)*

**Required Materials**

- Smartphone that can take photos and send photos via email
- Tuberculin syringe

**Important Notes**

- Local guidelines for privacy, use of phones for photography, and secure email transfer of photos should be followed.
- Healthcare workers should wear gloves during TST administration and while taking the mTST photo
- mTST photo should be taken within 1 minute of tuberculin injection

**Steps**

1. Prior to tuberculin injection, ask the patient for verbal agreement to take a photo of the tuberculin injection site. Patient agreement should be documented in the chart. All local standards for consent and documentation should be followed.
2. Prepare the patient for the mTST photos.

- The patient should be seated in the same position for tuberculin injection and mTST injection photos. Their arm should be resting on a table.
- The patient should remove any jewellery or watches on the arm that has been used for tuberculin injection.
- Position yourself directly in front of the patient when taking photos.

1. Perform TST administration as per standard practice.
2. Immediately following tuberculin injection, place an empty tuberculin syringe* between the tuberculin injection site and the patient’s elbow. It should be approximately “one thumb” distance (2 cm) behind the injection site. See Figure 1.

• If there is any blood around the tuberculin injection site it should be carefully wiped away with sterile gauze before the injection site is photographed.

** Syringe that was used for TST administration can be used as long as the needle has been disposed of.*

Figure 1. Placement of tuberculin syringe.


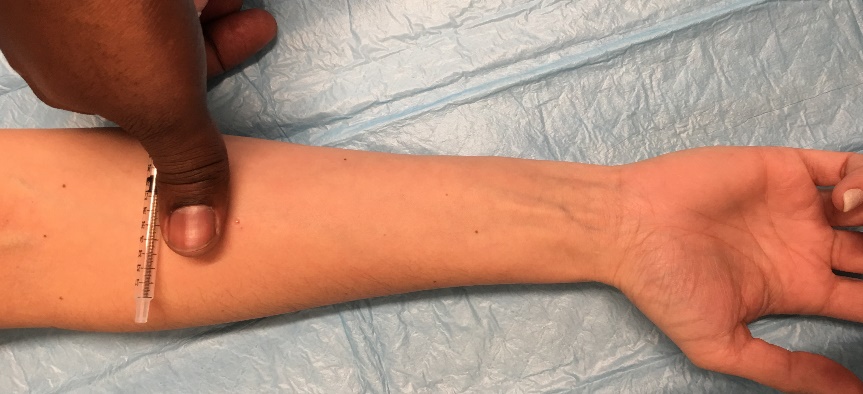


**Injection site**

1. Ask the patient to hold the syringe in place (using the arm that did NOT have the tuberculin injection).

- Turn the syringe so that tick marks on the syringe face the patient’s hand.
- Ensure the patient is holding the syringe level across their forearm (See Figure 2 for correct placement and Figure 3 for incorrect placement).

Figure 2. Level Syringe-CORRECT

**
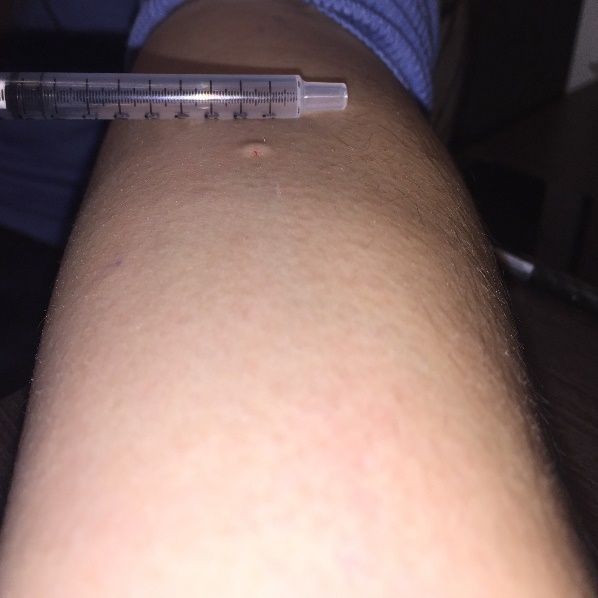
**

Figure 3. Tilted Syringe-INCORRECT


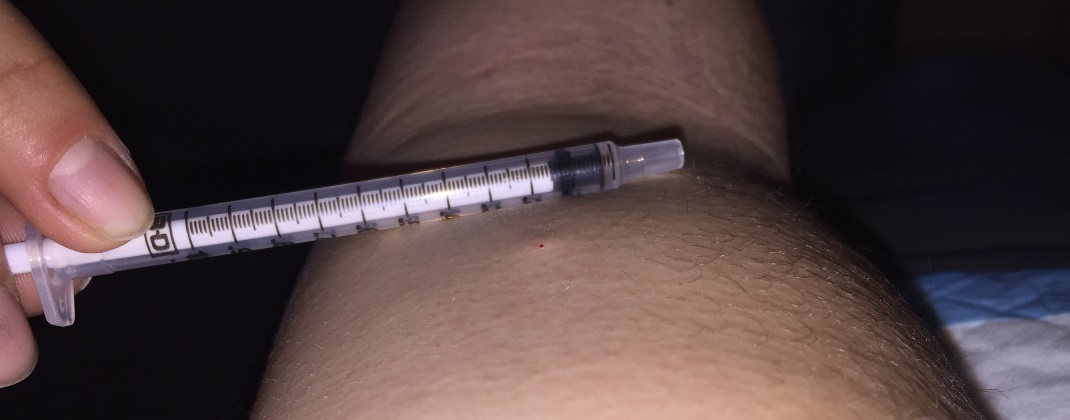


1. Open the camera application on your smartphone and **turn on the flash**.
2. Your phone should be placed approximately “one phone distance” (lengthwise) distal to the injection site when taking photos. See Figure 5 for an example of how to measure “one phone distance”. *Make sure your phone does not come into contact with any blood or tuberculin on the patient’s arm.*

Figure 4. Demonstration of how to measure “one phone distance” to determine where phone should be placed for photography of injection site.


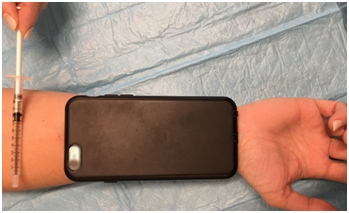


**Place phone here for taking photo**

**Injection site**

1. Position your phone as shown in Figure 5.

- Position the phone horizontally
- If the camera lens and flash are on one edge of the phone, the phone should be positioned so that the edge of the phone with the lens and flash is closer to the ceiling than the patient’s forearm (see Figure 5 for correct positioning and Figure 6 for incorrect positioning)
- For phones with the camera lens and flash in the middle of the phone, the phone should be orientated so that the flash is the furthest distance from the surface of the arm (camera flash is above lens). See Figure 7.

Figure 5. **Correct** camera lens and Figure 6. **Incorrect** camera lens and

flash orientation flash orientation


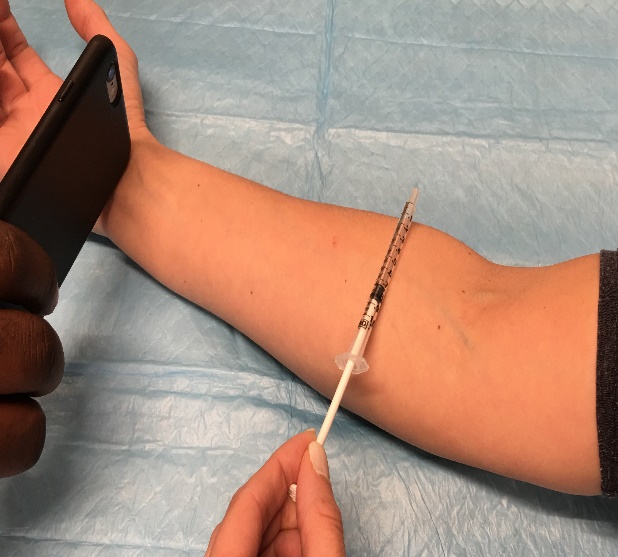

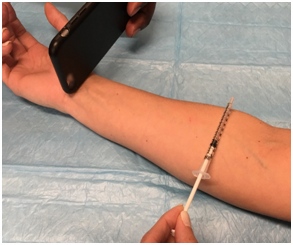


Figure 7. Phone and flash orientation located in middle of phone.


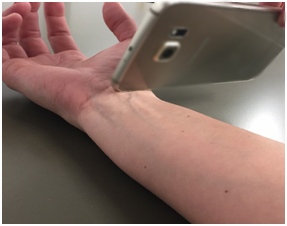


**Camera flash ABOVE lens**

1. Angle the smartphone towards the injection site until you can see the injection site and syringe together on the screen (Figure 8).

- The tick marks on the syringe should be visible in the photo.
- The patient’s elbow crease should be seen at the top of the screen.
- The patient’s arm should centered in the middle of the screen.

Figure 8. Correct positioning of phone.


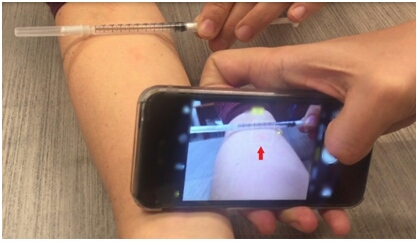


**Injection bleb. Tap here on phone screen to focus the camera.**

**Visible tick marks**

1. Tap the spot on your phone screen (viewfinder) that overlies the injection site to focus the camera on this spot. This should bring both the injection site and syringe ticks into clear focus (see Figure 8).
2. Take two photos with the camera in this position (or more if there are problems with positioning or the tuberculin syringe tick marks are not in focus).
3. Move the phone 1 cm towards the injection site and repeat steps 9-11.
4. Review the photos. Photos should meet ALL criteria listed in Table 1. See Figure 9 for an example of a good quality photo.

Table 1. Criteria for good quality photo

| **Criteria for good quality photo** |
| --- |
| - Syringe tick marks are in focus (ticks clearly seen- you may need to zoom in to check this) - Injection site is in focus (clearly seen) - Syringe is level across the arm - Patient’s arm is in the center of the photo - No patient identifying features (e.g. tattoos or jewellery) - Photo is well lit (i.e. flash is on) |

Figure 9. Good quality photo.

**
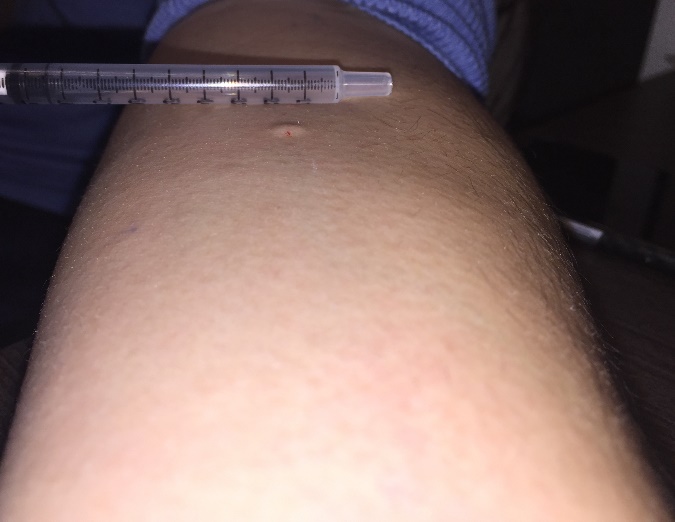
**

1. If you do not have any photos that meet ALL criteria in table 1 then take additional photos as needed.
2. Email the best three photos (based on the criteria listed in Table 1) to the mTST reviewer. Ensure you are following any local guidelines for electronic (email) transfer of data when emailing photos.

- The date the photos were taken should be listed in the email subject.
- **Do not email any patient information.**
- Photos should be sent as “Actual Size”. Do not reduce the size or resolution of the photos.

EMAIL ADDRESS of mTST reviewer: ______________________________________________

1. Delete the photos from your phone and the sent email from your email account after they have been emailed to the mTST reviewer.
