# Supplementary material for: The mTST – An mHealth approach for training and quality assurance of tuberculin skin test administration and reading
Source: PLoS One. 2019 Apr 17;14(4):e0215240. doi: 10.1371/journal.pone.0215240 (PMC6469794; doi:10.1371/journal.pone.0215240)

**Figure A. Example of tuberculin injection site photo.** A) Photo with adequate technical method, B) Photo with inadequate technical method- blurry photo, plus syringe too far away. C) Photo with inadequate technical method - injection site is not clearly visible because photo taken without flash, plus syringe too far away. D) Photo with inadequate technical method – syringe is tilted which makes measurement inaccurate.


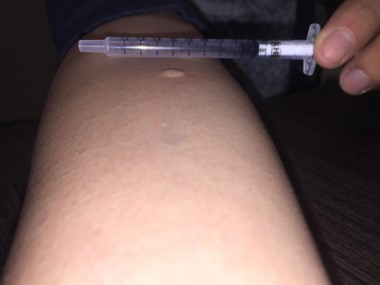

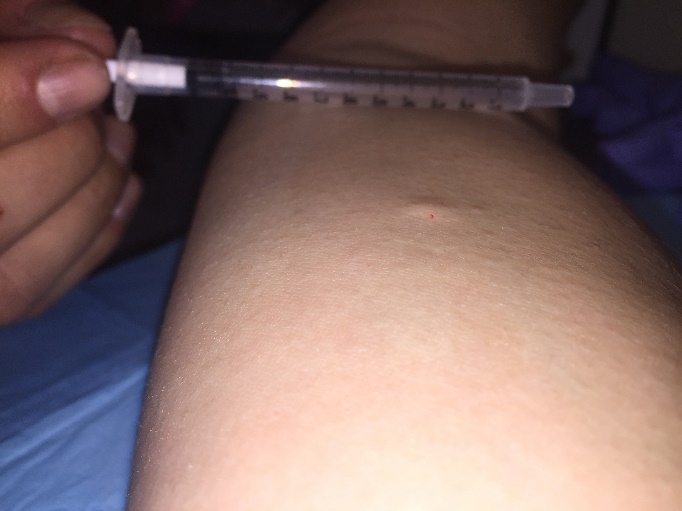


**B**

**A**


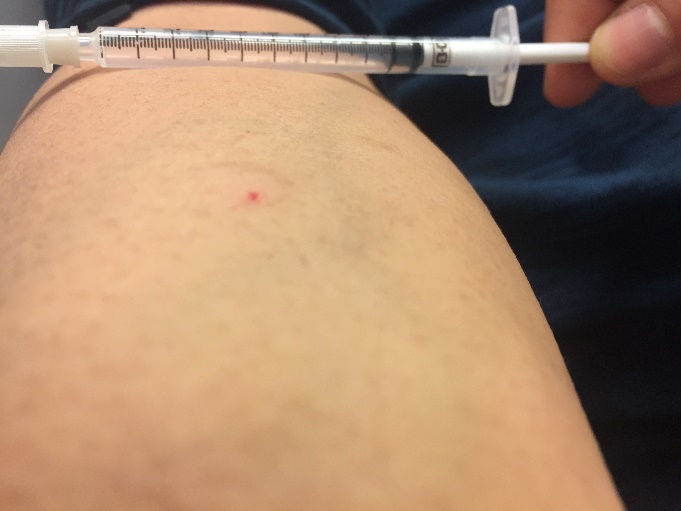

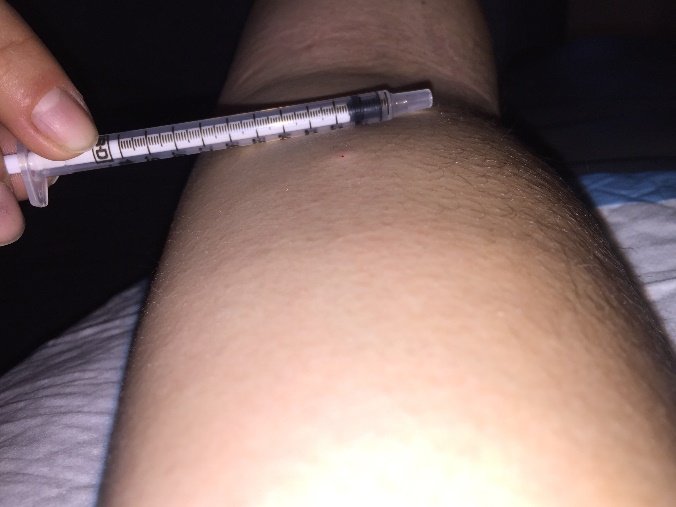


**C**

**D**

**Figure B. Examples of photos of TST induration**. A) Onsite measurement: 70 mm B) Onsite measurement: 16 mm C) Onsite measurement: 4 mm D) Onsite measurement: 37 mm


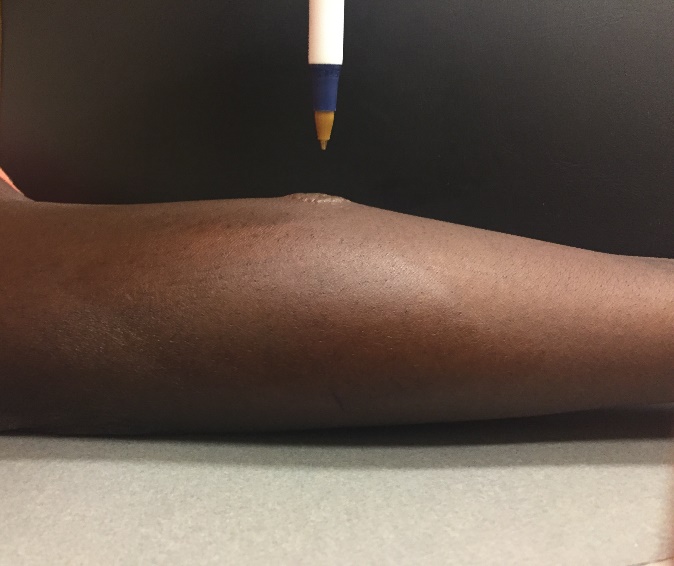

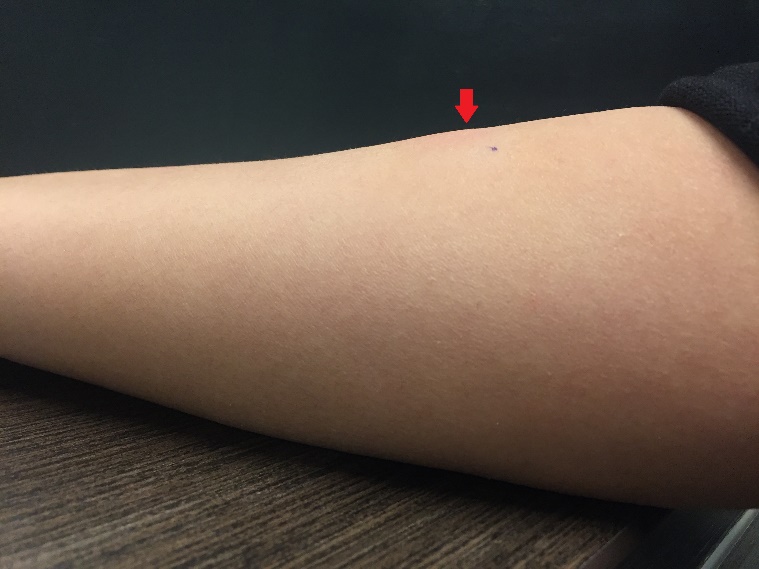


**A**

**B**


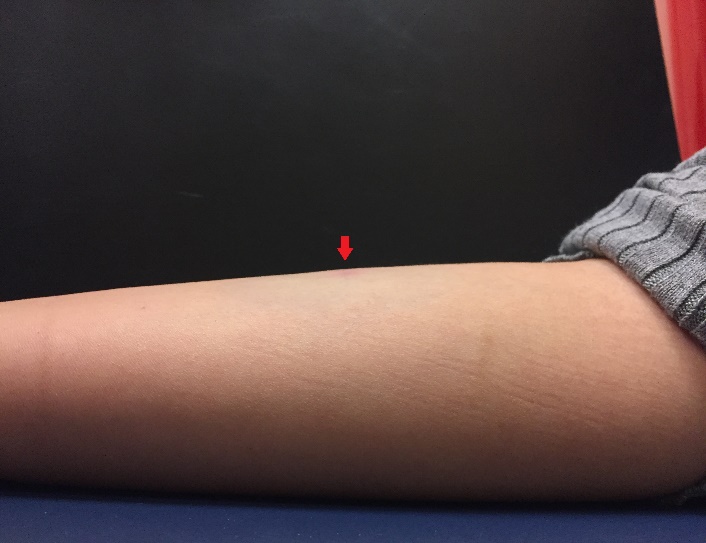


**C**

**C**

**
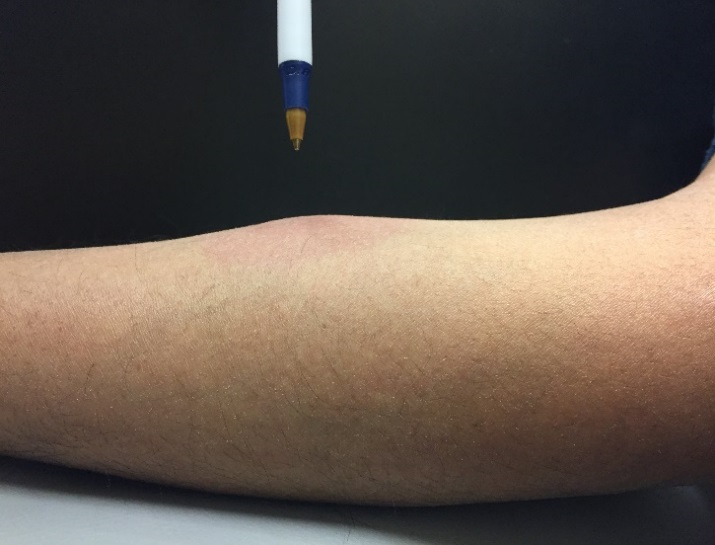
**

**D**

**Figure C. Onsite measurement of TST injection bleb.**


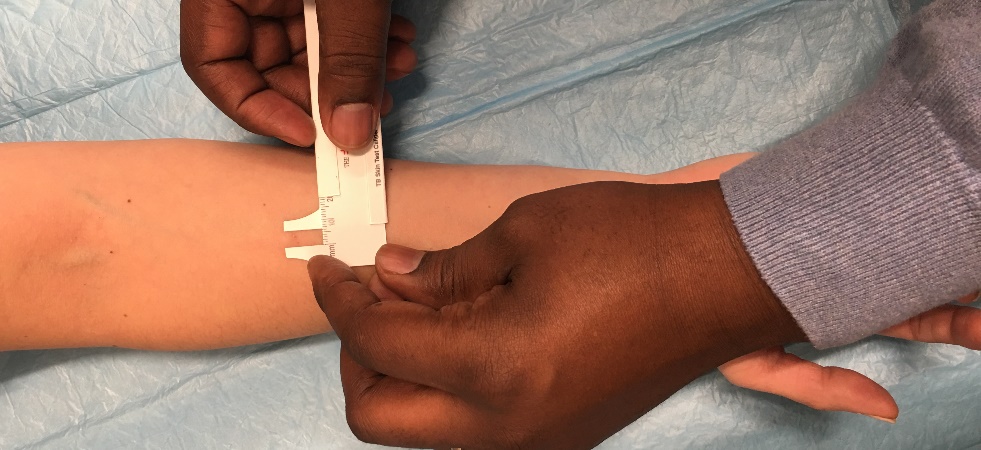

Supplement: S2 Appendix — (DOCX) [file pone.0215240.s002.docx]
