# Supplementary material for: The mTST – An mHealth approach for training and quality assurance of tuberculin skin test administration and reading
Source: PLoS One. 2019 Apr 17;14(4):e0215240. doi: 10.1371/journal.pone.0215240 (PMC6469794; doi:10.1371/journal.pone.0215240)
Supplement: S3 Appendix — (DOCX) [file pone.0215240.s003.docx]

*(This guide has two parts: First is a set of instructions for the reviewers to follow when actually performing a review of some mTST sent by a front-line Health care worker. This should be read as part of initial training of reviewers when first implementing the TST injection Quality assurance program. The 2^nd^ part – is also for initial training of the reviewers – to ensure there is standardization of quality assurance.)*

Guide prepared by: Saeedeh Moayedi-Nia, Leila Barss, Olivia Oxlade and Dick Menzies. of the McGill International TB Centre. If questions please contact: [olivia.oxlade@mcgill.ca](mailto:olivia.oxlade@mcgill.ca), or, [dick.menzies@mcgill.ca](mailto:dick.menzies@mcgill.ca). Montreal, August 2018

Note: The training video can be found at the following link: https://www.youtube.com/watch?v=DZbuygias7w&feature=em-share_video_user

**Part 1: Instructions for reviewers (responsible for TST quality assurance)**

These instructions describe how to review a mobile TST (mTST) photo of a TST injection site that has been taken by a healthcare worker immediately after the tuberculin injection.

**Required Materials**

- Computer that can receive photos sent via email
- Microsoft Paint or Mac Preview
- mTST Quality Assurance Table (see below)

**Getting started:**

Healthcare workers will email three mTST photos for each patient to the reviewer. Photos should contain the date the photos were taken as the subject header. There should be no patient identifying information in the email.

The reviewer will download the mTST photos that have been sent via email and evaluate the photos.

**Evaluation (Quality assurance)**

Evaluation is divided into three steps.

1. Quality of photo
2. Quality of TST injection
3. Size of TST injection ‘bleb’

**Step 1: Evaluate the quality of the photo.** In order to evaluate the TST injection properly, the photo must of good quality. The required criteria for a “good quality” mTST photo are listed in Table 1. The photo must meet ALL criteria to be considered “good quality”. Figure 1 is an example of a “good quality” photo.

Table A. Criteria required for “good quality” mTST photo

| **Criteria** |
| --- |
| 1. Syringe tick marks are in focus (clearly seen) *you may need to zoom in to check this 2. Injection site is in focus (clearly seen) 3. Syringe is level on arm (not tilted) 4. Syringe appears to be approximately “one thumb distance” or 2 cm behind the injection site 5. No identifying features in photo (e.g. tattoo or jewelry) 6. Photo is well lit (i.e. flash is on) |

**All criteria must be met to be considered a “good quality” photo*

Figure 1. “Good quality” mTST photo

**
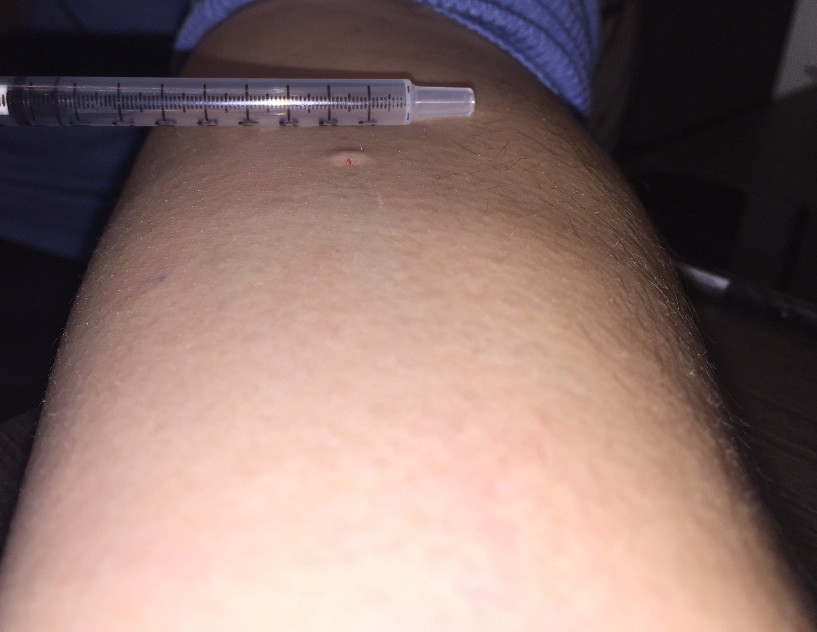
**

**Injection site in focus**

**Level syringe and visible ticks**

If all criteria are met, then record the photo as “good quality” in the first column of mTST Quality Assurance Table. If any of the criteria for a “good quality” TST photo are not met, then record this photo as “poor quality”. Record each criterion that has not been met and if necessary provide feedback on how to improve the photo. See Table 2 for an example.

Table B. mTST Quality Assurance Results – Example

| Photo Number | **Photo Quality**  **Step1**  (good quality/poor quality) | **Injection Quality**  Qualitative  **Step 2**  (good quality/poor quality) | **Injection Quality**  Quantitative  **Step 3**  (Size in mm, and quality assessment) | **Final Assessment**  **Step 4**  (correct/incorrect) |
| --- | --- | --- | --- | --- |
|  | **Criteria** | | |  |
|  | -Syringe tick marks in focus  -Injection site in focus  -Syringe is level  -Syringe location  -No identifying features | -Injection location  -Little or no leakage  -Minimal or no bleeding | 7 mm or greater = good quality  Less than 7mm = poor quality |  |
| 1 | Poor quality – syringe tilted |  |  |  |
| 2 | Good quality |  |  |  |
| 3 | Poor quality – Out of focus |  |  |  |
| 4 | Good quality |  |  |  |

**Step 2: Evaluate the quality of the TST injection.** This is divided into a qualitative evaluation and a quantitative evaluation. The qualitative criteria for a “good quality” injection are listed in Table 3.

Table C. Qualitative Criteria for TST injection

| **Qualitative Criteria** |
| --- |
| - Injection is located in the middle of the inner aspect of the forearm - Little or no leakage of injected tuberculin fluid - Minimal or no bleeding |

Perform the qualitative evaluation of the mTST injection first. If all qualitative criteria are met, then record “good quality” in the second column of TST Injection Quality Assurance Table. **If any of the qualitative criteria for a “good quality” TST injection are not met, then record this injection as “poor quality”.** Record each criterion that has not been met. See Figure 3 for an example of an injection that does not meet the criteria for “good quality”. See Table 4 for an example.

Figure 3. Example of Injection that does not meet qualitative criteria


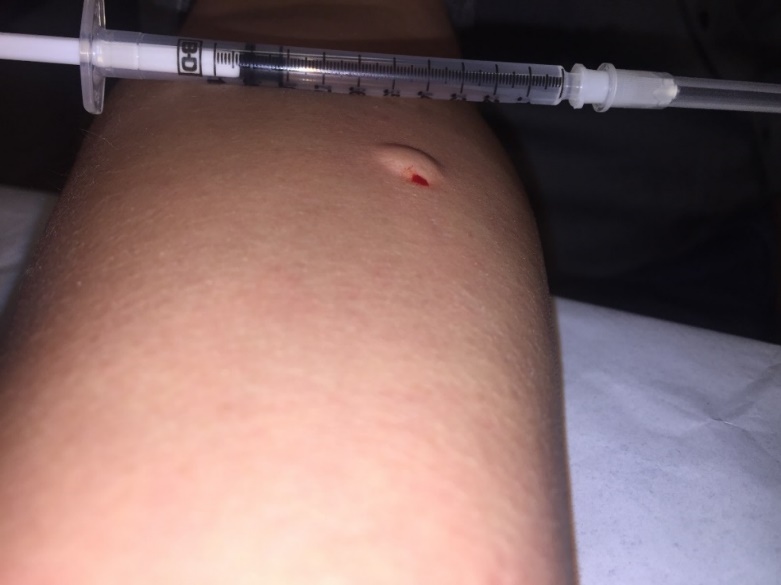


Injection is NOT located in middle of forearm

Table D. mTST Quality Assurance Results: Example

| Photo Number | **Photo Quality**  **Step1**  (good quality/poor quality) | **Injection Quality**  Qualitative  **Step 2**  (good quality/poor quality) | **Injection Quality**  Quantitative  **Step 3**  (Size in mm, and quality assessment) | **Final Assessment**  **Step 4**  (correct/incorrect) |
| --- | --- | --- | --- | --- |
|  | **Criteria** | | |  |
|  | -Syringe tick marks in focus  -Injection site in focus  -Syringe is level  -Syringe location  -No identifying features | -Injection location  -Little or no leakage  -Minimal or no bleeding | 7 mm or greater = good quality  Less than 7mm = poor quality |  |
| 1 | Poor quality– syringe tilted | NA (photo poor quality) |  |  |
| 2 | Good quality | Poor quality -Injection in wrong location |  |  |
| 3 | Poor quality – Out of focus | NA (photo poor quality) |  |  |
| 4 | Good quality | Good quality |  |  |

If the photo quality AND TST injection qualitative criteria are **both** judged as “good quality” then proceed to the next step-evaluating quantitative criteria. If the photo quality or TST injection qualitative criteria are judged to be “poor quality”, DO NOT COMPLETE THE QUANTITATIVE ASSESSMENT(Step3) and go to Step 4.

**STEP 3: Quantitative assessment of TST Injection**:

This assessment uses a validated method to ensure that the injection has been done properly. The steps are listed below with accompanying figures. The McGill team has also created a video that demonstrates these steps. (See  [video (link at start of document))](https://www.youtube.com/watch?v=DZbuygias7w&feature=em-share_video_user)

1. Open the photo in either the Preview (Mac OS X) or Microsoft paint (Windows).
2. Zoom in on the injection bleb.
3. Draw a thin black line on the injection bleb. The line should measure the diameter of the bleb at its widest transverse diameter (at a right angle to the long axis of the forearm).
4. Move the line next to the tuberculin syringe. Do not rotate the line.
5. Count the number of ticks the line covers.
6. Move the line back to the bleb and place in the location you used to measure the injection bleb diameter.
7. Write the number of ticks that were counted (that the line covered) in a text box beside the line.
8. Convert the number of ticks to millimeters using Table 5 below (this table is applicable for 1 ml TB syringe-27G x ½ needle). Please record the millimeter measurement in the Quantitative column of the mTST Quality Assurance Table.


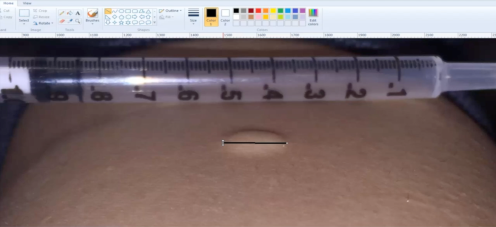


Figure 4. Step iii. Draw line.


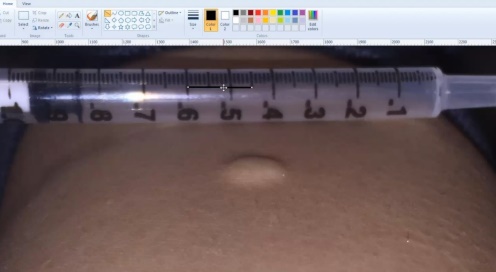


Figure 5. Step iv. Move line to tick marks


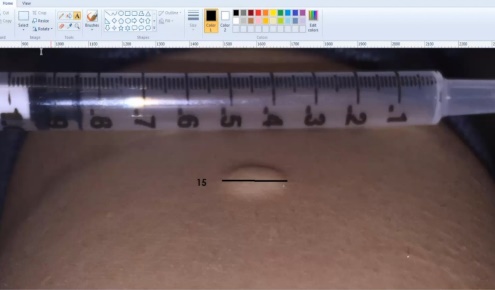


Figure 6. Step vii. Record measurement

Table E. Example: Conversion of syringe measurement to millimeters *(Note: this conversion chart applies to the syringes used in Montreal – this conversion chart will need to be verified and adapted, if needed, in all settings using this method)*

| Number of Syringe tick marks | mm |
| --- | --- |
| 5 | 3 |
| 6 | 3.5 |
| 7 | 4 |
| 8 | 4.5 |
| 9 | 5 |
| 10 | 5.5 |
| 11 | 6 |
| 12 | 7 |
| 13 | 7.5 |
| 14 | 8 |
| 15 | 8.5 |
| 16 | 9 |
| 17 | 9.5 |
| 18 | 10 |
| 18.5 | 10.5 |
| 19 | 11 |
| 20 | 11.5 |
| 21 | 12 |

Determine if the TST injection bleb size meets the quantitative criteria for “good quality”, meaning that this is a bleb of 7 mm or greater. Record the size in mm of the TST injection bleb in the third column of the mTST Quality Assurance Table, and if the injection is considered “good quality” or “poor quality” (See table 6)

Table F. mTST Quality Assurance Results. Example

| Photo Number | **Photo Quality**  **Step1**  (good quality/poor quality) | **Injection Quality**  Qualitative  **Step 2**  (good quality/poor quality) | **Injection Quality**  Quantitative  **Step 3**  (Size in mm, and quality assessment) | **Final Assessment**  **Step 4**  (correct/incorrect) |
| --- | --- | --- | --- | --- |
|  | **Criteria** | | |  |
|  | -Syringe tick marks in focus  -Injection site in focus  -Syringe is level  -Syringe location  -No identifying features | -Injection location  -Little or no leakage  -Minimal or no bleeding | 7 mm or greater = good quality  Less than 7mm = poor quality |  |
| 1 | Poor quality-syringe tilted | NA (photo poor quality) | NA (Failed qualitative assessment) |  |
| 2 | Good quality | Poor quality -Injection in wrong location | NA (Failed qualitative assessment) |  |
| 3 | Poor quality – Out of focus | NA (photo poor quality) | NA (Failed qualitative assessment) |  |
| 4 | Good quality | Good quality | 8mm – good quality |  |

**STEP 4:** Complete the final column on the mTST Quality Assurance Table “Final assessment” based on the following three parameters (See table 7):

1)Photo quality

2)TST Injection - qualitative criteria

3)TST Injection - quantitative criteria

- If ALL parameters are judged to be “good quality” 🡪 Mark as “CORRECT”
- If ANY parameter is judged to be “poor quality” 🡪 Mark as “INCORRECT

Table G. mTST Quality Assurance Results. Example

| Photo Number | **Photo Quality**  **Step1**  (good quality/poor quality) | **Injection Quality**  Qualitative  **Step 2**  (good quality/poor quality) | **Injection Quality**  Quantitative  **Step 3**  (Size in mm, and quality assessment) | **Final Assessment**  **Step 4**  (correct/incorrect) |
| --- | --- | --- | --- | --- |
|  | **Criteria** | | |  |
|  | -Syringe tick marks in focus  -Injection site in focus  -Syringe is level  -Syringe location  -No identifying features | -Injection location  -Little or no leakage  -Minimal or no bleeding | 7 mm or greater = good quality  Less than 7mm = poor quality |  |
| 1 | Poor quality-syringe tilted | NA (photo poor quality) | NA (Failed qualitative assessment) | Incorrect |
| 2 | Good quality | Poor quality -Injection in wrong location | NA (Failed qualitative assessment) | Incorrect |
| 3 | Poor quality – Out of focus | NA (photo poor quality) | NA (Failed qualitative assessment) | Incorrect |
| 4 | Good quality | Good quality | 8mm – good quality | Correct |

Feedback can be provided to the healthcare worker who submitted the photos either via email using the mTST Quality Assurance Table or verbally.

Generally, once a healthcare worker has submitted photos from 15 patients that have a final assessment as “CORRECT”, they are considered to have demonstrated reliable and appropriate TST administration technique. They do not need to continue to take photos on a regular basis. However, a program may decide to repeat the mTST quality assurance periodically – such as every 6 months or annually.

**Part 2: Training – review of mTST Photos**

This part of the training of QA reviewers consists of independently reviewing sets of mTST photos that have been selected to represent various potential qualitative and quantitative issues. After reading Part 1 of this guide, (above), the reviewer being trained should review the first set of 10 photos (*See Standard mTST photos: Part 1*) and complete the mTST quality assurance table (Blank copy – see Table 8 below) below. Their evaluations should be reviewed by their supervisor against the ‘answers’ for each photo given in Table 9 below. Any evaluations that are not concordant with the correct evaluations should be reviewed and discussed by supervisor and trainee-reviewer.

When the supervisor and trainee-reviewer are satisfied, and the trainee-reviewer achieves at least 90% concordance between their evaluations and the ‘answers’ then the trainee will review the second set of 10 mTST photos (*see Standard mTST photos: Part 2*) and compete a second mTST evaluation table. The reviewer’s evaluation can then be compared with the correct evaluation (Correct evaluations are shown in Table 10). We recommend that the reported quantitative measurement of the TST injection bleb fall within 2 mm of the measurement listed in the Training sets. If the reviewer’s measurements fall outside of this range, then their technique for measuring the TST injection bleb should be assessed.

Training will be completed when the trainee reviews the third set of 10 mTST photos (*Standard mTST photos: Part 3)* and achieves at least 90% concordance between evaluations and correct evaluations (Table 11).

Table H. mTST Quality Assessment -Evaluations for mTST Training BLANK

| Photo Number | **Photo Quality**  **Step1**  (good quality/poor quality) | **Injection Quality**  Qualitative  **Step 2**  (good quality/poor quality) | **Injection Quality**  Quantitative  **Step 3**  (Size in mm, and quality assessment) | **Final Assessment**  **Step 4**  (correct/incorrect) |
| --- | --- | --- | --- | --- |
|  | **Criteria** | | |  |
|  | -Syringe tick marks in focus  -Injection site in focus  -Syringe is level  -Syringe location  -No identifying features | -Injection location  -Little or no leakage  -Minimal or no bleeding | 7 mm or greater = good quality  Less than 7mm = poor quality | All STEPS1-3 criteria met |
|  |  |  |  |  |
|  |  |  |  |  |
|  |  |  |  |  |
|  |  |  |  |  |
|  |  |  |  |  |
|  |  |  |  |  |
|  |  |  |  |  |
|  |  |  |  |  |
|  |  |  |  |  |

Table I. mTST Quality Assessment -CORRECT Evaluations for mTST (for trainer only)-Training Set #1

| Photo Number | **Photo Quality**  **Step1**  (good quality/poor quality) | **Injection Quality**  Qualitative  **Step 2**  (good quality/poor quality) | **Injection Quality**  Quantitative  **Step 3**  (Size in mm, and quality assessment) | **Final Assessment**  **Step 4**  (correct/incorrect) |
| --- | --- | --- | --- | --- |
|  | **Criteria** | | |  |
|  | -Syringe tick marks in focus  -Injection site in focus  -Syringe is level  -Syringe location  -No identifying features | -Injection location  -Little or no leakage  -Minimal or no bleeding | 7 mm or greater | as comment above |
| 1 | Good quality | Good quality | 7mm | Correct |
| 2 | Poor quality- ticks not visible | Good quality | Not applicable | Incorrect |
| 3 | Good quality | Poor quality-Incorrect location | Not applicable | Incorrect |
| 4 | Good quality | Good quality | 8mm | Correct |
| 5 | Good quality | Good quality | 9mm | Correct |
| 6 | Good quality | Poor quality-Incorrect location | Not applicable | Incorrect |
| 7 | Good quality | Good quality | 5.5mm | Incorrect |
| 8 | Poor quality-photo not in focus | Good quality | Not applicable | Incorrect |
| 9 | Good quality | Good quality | 10mm | Correct |
| 10 | Good quality | Good quality | 4mm | Incorrect |

Table J. mTST Quality Assessment -CORRECT Evaluations for mTST (for trainer only) -Training Set #2

| Photo Number | **Photo Quality**  **Step1**  (good quality/poor quality) | **Injection Quality**  Qualitative  **Step 2**  (good quality/poor quality) | **Injection Quality**  Quantitative  **Step 3**  (Size in mm, and quality assessment) | **Final Assessment**  **Step 4**  (correct/incorrect) |
| --- | --- | --- | --- | --- |
|  | **Criteria** | | |  |
|  | -Syringe tick marks in focus  -Injection site in focus  -Syringe is level  -Syringe location  -No identifying features | -Injection location  -Little or no leakage  -Minimal or no bleeding | 7 mm or greater |  |
| 11 | Good quality | Poor quality-Incorrect location | Not applicable | Incorrect |
| 12 | Good quality | Good quality | 8mm | Correct |
| 13 | Good quality | Poor quality-Incorrect location | Not applicable | Incorrect |
| 14 | Poor quality-ticks not visible | Good quality | Not applicable | Incorrect |
| 15 | Good quality | Good quality | 10.5mm | Correct |
| 16 | Poor quality-ticks not visible | Good quality | Not applicable | Incorrect |
| 17 | Good quality | Good quality | 5mm | Incorrect |
| 18 | Good quality | Good quality | 5mm | Incorrect |
| 19 | Good quality | Good quality | 8mm | Correct |
| 20 | Good quality | Good quality | 5mm | Incorrect |

Table K. mTST Quality Assessment - CORRECT Evaluations for mTST (for trainer only) - Training Set #3

| Photo Number | **Photo Quality**  **Step1**  (good quality/poor quality) | **Injection Quality**  Qualitative  **Step 2**  (good quality/poor quality) | **Injection Quality**  Quantitative  **Step 3**  (Size in mm, and quality assessment) | **Final Assessment**  **Step 4**  (correct/incorrect) |
| --- | --- | --- | --- | --- |
|  | **Criteria** | | |  |
|  | -Syringe tick marks in focus  -Injection site in focus  -Syringe is level  -Syringe location  -No identifying features | -Injection location  -Little or no leakage  -Minimal or no bleeding | 7 mm or greater |  |
| 21 | Poor quality- ticks not visible | Not applicable | Not applicable | Incorrect |
| 22 | Good quality | Good quality | 8.5mm | Correct |
| 23 | Good quality | Good quality | 10mm | Correct |
| 24 | Good quality | Poor quality -Incorrect location | Not applicable | Incorrect |
| 25 | Good quality | Good quality | 3mm | Incorrect |
| 26 | Poor quality - Bleb not visible | Not applicable | Not applicable | Incorrect |
| 27 | Good quality | Poor quality- leakage | Not applicable | Incorrect |
| 28 | Good quality | Good quality | 8.5mm | Correct |
| 29 | Poor quality - Bleb not visible | Poor quality -Incorrect location | Not applicable | Incorrect |
| 30 | Good quality | Good quality | 5mm | Incorrect |
